# Direct Detection of Rifampin-Resistant *Mycobacterium tuberculosis* in Respiratory Specimens Using Quantamatrix Multiplexed Assay Platform (QMAP) System: A Multicenter Study in Korea

**DOI:** 10.3389/fmicb.2018.01804

**Published:** 2018-08-17

**Authors:** Hye-young Wang, Kwangjin Ahn, Young Uh, Hyeyoung Lee, Seoyong Kim, Yunhee Chang, Chulhun L. Chang, Tae-sun Shim

**Affiliations:** ^1^Optipharm, Inc., Wonju Eco Environmental Technology Center, Wonju-si, South Korea; ^2^Department of Laboratory Medicine, Yonsei University Wonju College of Medicine, Wonju-si, South Korea; ^3^Department of Biomedical Laboratory Science, College of Health Sciences, Yonsei University, Wonju-si, South Korea; ^4^Department of Laboratory Medicine, Pusan National University Yangsan Hospital, Yangsan, South Korea; ^5^Division of Pulmonary and Critical Care Medicine, Department of Internal Medicine, University of Ulsan College of Medicine, Asan Medical Center, Seoul, South Korea

**Keywords:** *Mycobacterium tuberculosis*, rifampin, drug resistance, nucleic acid amplification techniques, Quantamatrix Multiplexed Assay Platform

## Abstract

Rapid and accurate detection of rifampin-resistant *Mycobacterium tuberculosis* (MTB) is of primary importance for infection control and selection of anti-tuberculosis drugs. The aim of this study was to evaluate the usefulness of a newly developed multiplexed, bead-based bioassay (Quantamatrix Multiplexed Assay Platform, QMAP) for the direct detection of rifampin-resistant MTB in respiratory specimens. A total of 400 respiratory specimens collected from patients with clinically suspected tuberculosis or non-tuberculous mycobacteria (NTM) infections were tested with the culture-based conventional *Mycobacterium* species identification and QMAP system. Among 400 specimens, 154 samples were evaluated using phenotypic anti-tuberculosis drug susceptibility test (DST) and the QMAP system for the detection of rifampin resistance. Detection agreement rate between the culture-based conventional identification and QMAP system for MTB and NTM according to acid-fast bacillus smear positivity was as follows: 97.0% (131/135) and 93.6% (88/94) in 229 smear-positive samples and 69.4% (25/36) and 73.0% (65/89) in 171 smear-negative samples. Based on culture as the gold standard, the overall sensitivity and specificity of the QMAP system for *Mycobacterium* identification were 87.3 and 97.8%, respectively. The categorical agreement rate between phenotypic DST and QMAP system for rifampin was as follows: complete agreement, 92.9% (143/154); very major error, 0%; and major error, 0.6% (1/154). The overall sensitivity of the QMAP system for the detection of rifampin resistance was 97.1% (34/35). The QMAP system is a useful screening method for the early diagnosis of tuberculosis and selection of anti-tuberculosis drug, as it may detect rifampin-resistant MTB directly from respiratory specimens.

## Introduction

Tuberculosis (TB) is associated with significant mortality and morbidity and is still considered a deadly disease worldwide (Zaman, 2010). The World Health Organization (WHO) reported 10.4 million new TB cases and 1.4 million TB-related deaths in 2015 (World Health Organization, 2016). South Korea, in particular, has an intermediate TB burden and a higher TB prevalence than other developed countries. Although the prevalence of TB and new infections has decreased, the incidence of non-tuberculous mycobacteria (NTM) is increasing worldwide (World Health Organization, 2016; Yoon et al., 2017). Species identification of NTM is recommended because the clinical significance and drug resistance patterns of NTM differ per species (Maurya et al., 2012; Thanachartwet et al., 2014). The most effective means of protection against mycobacterial infections is early diagnosis and treatment. TB treatment and control has been severely compromised in recent years, owing to the increase in the prevalence of drug-resistant TB (Abubakar et al., 2013).

As per the WHO estimation, the global burden of multidrug-resistant TB (MDR-TB), defined as TB with resistance to both rifampin (RIF) and isoniazid, increased from 500,000 cases per year to nearly 2,000,000 cases in 2015 (World Health Organization, 2016). Standard identification and drug resistance assays for mycobacterial infections are cumbersome, labor-intensive, and time-consuming. The detection of drug-resistant TB with the results of a drug susceptibility test (DST) may take weeks to months and this delay may unnecessarily lead to inadequate drug regimens, worsening of the disease state, and further transmission of MDR-TB.

Various molecular techniques are available for the detection of anti-TB drug resistance: electrophoresis-based techniques, PCR-DNA sequencing, hybridization-based techniques, real-time PCR, multiplex allele-specific PCR, line probe assay, nucleic acid amplification-based techniques, loop-mediated isothermal amplification technique, and whole genome sequencing (Garcia de Viedma, 2003; Noor et al., 2015; Park et al., 2018). The Xpert MTB/RIF assay (Cepheid, Sunnyvale, CA, United States) directly detects MTB and RIF resistance from untreated sputum with a minimal handling time of less than 2 h. The diagnostic performance is proved through testing respiratory specimens gathered from around the world (Boehme et al., 2010). The recently developed Quantamatrix Multiplexed Assay Platform (QMAP) system (QuantaMatrix, Seoul, South Korea) using a multiplexed, bead-based bioassay with shape-encoded silica microparticles (Kim et al., 2015) is being studied and evaluated. The QMAP diagnostic platform uses a specifically encrypted magnetic microdisk technology. One disk is coated with 50 μm silica, and each disk has a magnetic property to prevent the loss of microdisks. As each magnetic microdisk has a unique shape, it is recognized as a graphic barcode. 5′-Amine target-specific oligonucleotide probes is easily immobilized on the carboxyl-functionalized microdisk. A single microwell system has been proven capable of 1000 multiplex capacities, which means that only one microwell is required to test 1000 types of pathogens in one sample. Twenty-three species of *Mycobacterium* that can be identified in QMAP are: the *Mycobacterium* genus, *Mycobacterium tuberculosis* complex (MTBC), *M. avium, M. intracellulare, M. scrofulaceum, M. abscessus* complex, *M. chelonae, M. fortuitum* complex, *M. ulcerans*/*M. marinum, M. kansasii*/*M. gastri, M. genavense*/*M. simiae, M. terrae, M. nonchromogenicum, M. celatum, M. gordonae, M. szulgai, M. mucogenicum, M. aubagnense, M. malmoense, M. smegmatis, M. phlei, M. xenopi, M. flavescens*, and *M. peregrinum*/*M. septicum*. Also, RIF resistance of MTBC and NTM can be confirmed by detecting *rpoB* gene mutation (Wang et al., 2017a,b,c).

In our previous study (Wang et al., 2017c), the QMAP system for the simultaneous detection of *M. tuberculosis* (MTB) and RIF resistance gene using cultured mycobacteria showed 100% sensitivity and 97.8% specificity for the identification of *Mycobacterium* species, and showed 100% concordance for RIF resistance gene detection as compared with the phenotypic anti-TB DST. In this study, we evaluated the ability of the QMAP system to rapidly and accurately detect RIF-resistant MTB using the respiratory specimens obtained from multi-centers in Korea.

## Materials and Methods

### Study Population

A total of 400 respiratory specimens archived after the routine exam for TB or NTM infection at three tertiary care hospitals (Pusan National University Yangsan Hospital, Asan Medical Center, and Wonju Severance Christian Hospital) located in South Korea were used to evaluate the efficacy of the QMAP test. The research was carried out from August 2016 to April 2017. When the patients were suspected of having a tuberculosis infection, their respiratory specimen was collected for clinical diagnosis. After proper tests were run, the residual samples were gathered. These samples were anonymized and unlinked before this study was conducted. The study was exempted from IRB review, according to government regulation, and the exemption approval was received from the Institutional Review Board of Wonju Severance Christian Hospital (approval no. CR316304), the Institutional Ethics Committee of Asan Medical Center (approval no. S2016-1326-0001), and Institutional Review Board of National University Yangsan Hospital (approval no. 04-2016-010). The samples were collected and sent to The Biomedical Laboratory Science (Yonsei University, College of Health Sciences, Wonju, South Korea) within 24 h under room temperature.

### Acid-Fast Bacillus (AFB) Smear, Culture, Conventional *Mycobacterium* Identification, and Phenotypic DST

To prepare the AFB smears, sputum specimens were decontaminated using *N*-acetyl cysteine (NALC)-2% sodium hydroxide (NaOH) method. Auramine-rhodamine fluorescent staining was performed on the sputum specimens pretreated with NALC-NaOH, and the results were confirmed using the Ziehl-Neelsen method (Kent, 1985). Microscopy results were obtained using semi-quantitative analysis. The presence of AFB in a specimen was defined as follows: trace = 1–2 AFB per 300× field; 1 + = 1–9 AFB per 100× field; 2 + = 1–9 AFB per 10× field; 3 + = 1–9 AFB per 1× field; and 4 + = more than 9 AFB per 1× field. The specimen with trace results in AFB smear was categorized as smear-negative. The NALC-NaOH-pretreated sputum specimens were inoculated onto solid agar (Ogawa medium; Asan Pharmaceutical, Seoul, South Korea) and liquid medium (mycobacteria growth indicator tube [MGIT]; BD Diagnostic Systems, Sparks, MD, United States). Cultures on Ogawa medium were incubated at 37°C under 5% CO_2_ for 8 weeks. Cultures in MGIT tubes were incubated using the BACTEC MGIT 960 system (BD Diagnostic Systems) and continuously monitored for 6 weeks. An aliquot (1 mL) from each MGIT culture was tested and those with positive signals were subjected to AFB staining to confirm the presence of AFB and exclude contamination. All positive culture isolates were sent to the Korean Institute of Tuberculosis (Osong, South Korea), which serve as a reference laboratory; conventional microbiological and biochemical tests were used for the identification and classification of mycobacteria. Phenotypic DST for the first- and second-line drugs was performed with the absolute concentration method on Löwenstein-Jensen medium-based M-KIT plates (The Korean Institute of Tuberculosis, Osong, South Korea) containing 12 anti-TB drugs. The isolates were tested for resistance to critical concentrations of RIF (40.0 μg/mL).

### DNA Preparation

To extract genomic DNA, one volume of respiratory specimens was first liquefied and one volume of 4% NaOH was added for digestion and decontamination. After washing with 40 μL of phosphate buffered saline (PBS, PH 8.0), it was incubated at room temperature for 30 min and centrifuged at 13,000 × *g* for 1 min. Supernatants were removed and pellets were re-suspended with 1.0 mL sterile distilled water (ddH_2_O) and centrifuged at 13,000 × *g* for 1 min. The washing step was repeated twice and the pellets were re-suspended in 100 μL DNA extraction solution (5% Chelex resin, Bio-Rad, United States).

### QMAP System

The QMAP system was used according to the manufacturer’s instructions. Each sample was tested in duplicates, and all QMAP test runs were performed twice. Twenty-four *Mycobacterium* genus- and species-specific oligonucleotide probes (Wang et al., 2017a), and 10 RIF probes were used. MTB-specific oligonucleotide probe was designed to distinguish between MTB and NTM according to 16S-23S rRNA internal transcribed spacer region of *Mycobacterium* species in NCBI database. Five of the 10 RIF probes are probes for the wild-type (WT) *rpoB* amino acids 509–534. Five mutant-type (MT) RIF probes that account for the most common specific mutations, including 513CAA(Gln)-CCA(Pro) (MT1), 516GAC(Asp)-TAC(Tyr) (MT2), 516GAC(Asp)-GTC(Val) (MT3), 526CAC(His)-TAC(Tyr) (MT4), and 531TCG(Ser)-TTG(Leu) (MT5), were also used.

The 5′-amine-modified carboxylated microdisks were coupled to unique probes, wherein each of the target *Mycobacterium* species genes was complementary to a single probe. In brief, PCR was performed using 20-μL reaction mixture (GeNet Bio, Daejeon, South Korea) containing 2× master mix, 1× biotinylated primer mixture, 5 μL of sample DNA, and ddH_2_O to make up a final volume of 20 μL. A total of 40 PCR cycles comprised an initial denaturation step at 95°C for 30 s, followed by annealing and extension at 65°C for 30 s. After the final cycle, the samples were maintained at 72°C for 10 min to complete the synthesis of all strands. The biotinylated PCR products were denatured at 25°C for 5 min in a denaturation solution, diluted in 45 μL of hybridization solution, and then added to the coupled microdisks in the provided glass MatriPlate (Brooks, Chelmsford, MA, United States). The denatured single-stranded PCR products were hybridized with the coupled probes on the microdisks for 30 min at 40°C. The microdisks were washed thrice with gentle shaking in 120 μL of washing solution for 1 min at 25°C and incubated with 1:2,000 diluted streptavidin R–phycoerythrin conjugate (ProZyme, San Leandro, CA, United States) in a conjugate diluent solution for 10 min at 25°C. Following incubation, the microdisks were washed thrice with 120 μL of washing solution for 1 min at room temperature. Bright-field and fluorescence microscopic images were obtained for data analysis. The fluorescence intensity of all the microdisks in an image was automatically measured using the provided software. The cutoff value to distinguish between the positive and negative results was the fluorescence intensity signal of 500 (Wang et al., 2017a). When a positive signal was seen from two or more probes in a specimen, the probe with a positive fluorescence intensity twice higher than that of the other probe was reported as positive. The time taken for DNA preparation and hybridization was at least 90 min. DNA preparation and hybridization were the only processes that required manual control. The time taken for bacterial detection was 30 s per sample (Wang et al., 2017c).

### Analysis of *rpoB* Gene Sequence

To confirm the identity of the samples with discrepant results between the conventional method and QMAP system for NTM identification, *rpoB* gene region was sequenced. The primer set used to amplify the target *rpoB* gene was 372F 5′-CCTGTTCTTCAAGGAGAAGCGCTACGACCTGG-3′ and 372R 5′-GGACGGATGTTGATCAGGGTCTGCGG-3′, which resulted in a 372-bp PCR product. The amplified DNA (*rpoB* region) was sequenced using ABI Prism BigDye Terminator kit and an ABI 3730 automated DNA sequencer (Cosmo Genetech, Seoul, South Korea). The sequences obtained were compared with the sequences in the National Center for Biotechnology Information (NCBI) GenBank database for species assignment.

### Comparison of Results

We analyzed interpretive categorical agreement between the phenotypic DST and QMAP system for RIF susceptibility. Complete categorical agreement was defined as the same RIF result with phenotypic DST and QMAP system. RIF categorical error rates were calculated and reported as follows: a very major error was recorded if an isolate was found to be susceptible on the QMAP system and resistant on the phenotypic DST, and a major error was recorded if an isolate was found to be resistant on the QMAP system and susceptible on the phenotypic DST method.

## Results

The number of AFB smear-positive cases was 299. The number of isolation MTB complex or NTM on conventional culture method was 354. The number of identifications of MTB complex or NTM on QMAP system was 355. The detection agreement rate between the conventional identification and QMAP system for MTBC and NTM was as follows: 97.0% (131/135) and 93.6% (88/94) in 229 AFB smear-positive samples and 69.4% (25/36) and 73.0% (65/89) in 171 AFB smear-negative samples, respectively. Based on culture as the gold standard, the overall sensitivity and specificity of the QMAP system for identification were 87.3 and 97.8%, respectively. Of the 153 samples that showed NTM-positive signals in the QMAP system, four were positive on both probes (two samples, MTB complex and *M. intracellulare*; two samples, MTB complex and *M. avium*). The *rpoB* sequence analysis of four samples showing two positive signals revealed the presence of *M. intracellulare* and *M. avium*. Among 46 culture-negative samples, one sample showed a positive signal for *M. celatum* by the QMAP system, which was confirmed as *M. celatum* by sequence analysis (**Table 1**).

**Table 1:** Detection agreement between the conventional method and QMAP system according to AFB smear.

AFB smear (*n*)	*Mycobacterium* species	Number of isolates by *Mycobacterium* identification method	
		Conventional method	QMAP system
Positive (229)	MTB complex	135	131
	NTM	94	88^a^
Negative (171)	MTB complex	36	25
	NTM	89	65^b^
	Negative	46^c^	45

*^a^Sequence analysis of two samples with two positive signals (MTB complex [fluorescence intensity {FI}, 1,008] and *Mycobacterium intracellulare* [FI 2,036]; MTB complex [FI 1,082] and *Mycobacterium avium* [FI 3,811]) revealed the presence of *M. intracellulare* and *M. aviu*m. ^b^Sequence analysis of two samples with two positive signals (MTB complex [FI, 787] and *M. intracellulare* [FI, 3,811]; MTB complex [FI, 1,214] and *M avium* [FI, 2,750]) revealed the presence of *M. intracellulare* and *M. avium*. ^c^One sample showed a positive signal for *Mycobacterium celatum* (FI, 1,040) by QMAP system. AFB, acid-fast bacillus; MTB, *Mycobacterium tuberculosis*; NTM, non-tuberculous mycobacteria.*

The QMAP system detected RIF resistance for 29/30 (96.7%) samples from 130 AFB smear-positive and culture-positive samples and 5/5 (100%) samples from 24 AFB smear-negative and culture-positive samples. One isolate showing RIF sensitivity by phenotypic DST and RIF resistance by the QMAP system was confirmed to carry a mutated codon 531TTG by sequence analysis. The overall sensitivity of the QMAP system for the detection of RIF resistance was 97.1% (34/35) (**Table 2**).

**Table 2:** Detection agreement between phenotypic anti-TB drug susceptibility test (DST) and QMAP system for rifampin resistance of 154 *Mycobacterium tuberculosis* (MTB) complex isolates.

AFB smear	Number of MTB isolates according to rifampin resistance detection method			
	Phenotypic DST		QMAP system	
			*rpoB* gene mutation not-detected	*rpoB* gene mutation detected
4+	Rifampin-S	31	31	0
	Rifampin-R	14	0	14
3+	Rifampin-S	20	20	0
	Rifampin-R	8^a^	0	7
2+	Rifampin-S	22	22	0
	Rifampin-R	8	0	8
1+	Rifampin-S	27	27	0
	Rifampin-R	0	0	0
Trace	Rifampin-S	9^b^	2	1^c^
	Rifampin-R	4	0	4
Negative	Rifampin-S	10^d^	7	0
	Rifampin-R	1	0	1

*^a^One isolate showed fluorescence intensity lesser than the cutoff value of QMAP system. ^b^Six isolates showed fluorescence intensity lesser than the cutoff value of QMAP system. ^c^Isolate was confirmed of harboring a mutated codon 531TTG by sequence analysis. ^d^Three isolates showed fluorescence intensity lesser than the cutoff value of QMAP system. AFB, acid-fast bacillus; S, susceptible; R, resistant.*

The complete categorical agreement, very major error, and major error between the QMAP system and phenotypic DST method for RIF resistance were 92.9, 0, and 0.6%, respectively. A total of 6.5% (10/154) samples showed fluorescence intensity less than the cutoff value of the QMAP system (**Table 3**).

**Table 3:** Interpretive category errors of rifampin determined by comparing QMAP system and phenotypic DST for 154 *Mycobacterium tuberculosis* complex isolates.

No. (%) in complete agreement	No. (%) of interpretive category discrepancies	
	Very major error	Major error
143 (92.9)	0 (0)	1^a^ (0.6)

*^a^Isolate was confirmed to harbor a mutated codon 531TTG by sequence analysis.*

## Discussion

Early detection of RIF resistance of MTB is important because the RIF-resistant MTB isolates are prone to resistance to other anti-TB drugs (Chien et al., 2015). Therefore, an accurate and rapid RIF susceptibility testing for MTB is desirable for appropriate anti-TB drug therapy and to monitor the further spread of MDR-TB. The rising prevalence of NTM in the South Korea has highlighted the necessity for the accurate differential identification of NTM from MTB and NTM species-level identification. The simultaneous detection of *Mycobacterium* species and RIF-resistant MTB was shown to improve patient outcomes (Johnson et al., 2008) and reduce hospital costs. Therefore, it is necessary to develop a reliable, rapid, and cost-effective diagnostic tool for the simultaneous detection of MTB and RIF resistance.

The core technology used in this study is a magnetic microdisk carrying different types of shaped-disks as graphically encoded carboxyl-functionalized microparticles on a glass plate developed from QuantaMatrix. Advantages of the technology include low cost, high chemical resistance, reduced nonspecific adsorption rates, and increased physical process tolerance such as sonication. These microdisks have stronger fluorescence than those employed in other assays using probes that react with the target sequence (Kim et al., 2015). The microdisk-based QMAP system hybridized with multiplex PCR and immobilized probes containing WTs and MTs showed good agreement with the phenotypic DST. The accuracy of the QMAP system improved after the calculation of the average fluorescence intensity of 12 fields for each microwell. Thus, the system provides an accuracy equivalent to or better than the conventional phenotypic methods and may easily distinguish between the bacterial species with similar phenotypic characteristics and exclude subjective interpretation (Wang et al., 2017b).

The application of the QMAP system for direct specimen evaluation proved its ability to identify MTB or NTM and simultaneously confirm RIF resistance. Teo et al. (2011) reported that the sensitivity and specificity of identification for respiratory specimens with Xpert MTB/RIF assay were 90.3 and 91.6%, respectively, while those for Gen-Probe’s Amplified MTD assay were 96.8 and 91.2%, respectively. On the other hand, the overall sensitivity and specificity of the QMAP system for *Mycobacterium* identification were, respectively, 87.3 and 97.8% in this study, suggesting that the results of the QMAP system are comparable to those of the Xpert MTB/RIF assay and Gen-Probe’s Amplified MTD assay. As for all tests, the diagnostic performance of the QMAP system for *Mycobacterium* identification may be different in settings with different TB prevalence.

There were two similar trials that aimed to detect the *rpoB* gene in direct sputum specimens. The sensitivity and specificity of PCR-based universal heteroduplex generator assay for the detection of RIF resistance in culture-positive specimens were 83 and 98.2%, respectively (Williams et al., 1998). Of the 64 AFB smear-positive and culture-positive sputum samples from retreatment TB cases in Uganda, 63 (98.4%) were detected and 9/9 (100%) were RIF resistant as revealed by Xpert MTB/RIF assay (Helb et al., 2010). In this study, the RIF resistance detection with the QMAP system identified 29/30 (96.7%) AFB smear-positive samples and 5/5 (100%) AFB smear-negative samples, suggesting that the efficiency of the QMAP system for RIF resistance detection is comparable to that of the Xpert MTB/RIF assay.

The Xpert MTB/RIF assay is rapid and easy, but the exact identification and differentiation of *Mycobacterium* at species level is not yet available (Boehme et al., 2010; Helb et al., 2010; Teo et al., 2011). Although the QMAP system needs more time and has a more complicated specimen preparation process, it could identify the exact species as well as RIF resistance. Since the threat of NTM infection is increasing, the QMAP system could be the choice for diagnosis of NTM infection.

In conclusion, the QMAP system provides a simple protocol that completed the identification process within 3 h and potentially shortened the time to reliably detect the RIF-resistant MTB in direct pulmonary specimens from patients with clinically suspected TB or NTM. Further validation studies with a larger number of samples and including clinical data of each patient would be warranted.

## Author Contributions

H-YW and KA contributed equally as first authors. YU and HL contributed equally as corresponding authors. SK and YC helped to generate the Quantamatrix Multiplexed Assay Platform (QMAP) system. CC and T-SS gathered respiratory specimens from theirs’ laboratory.

## Conflict of Interest Statement

The authors declare that the research was conducted in the absence of any commercial or financial relationships that could be construed as a potential conflict of interest.
